# Supplementary material for: “Healing hands: Understanding physiotherapists’ knowledge, attitudes and barriers on pressure injury prevention in the medical wards in Jordan”
Source: PLoS One. 2026 Mar 10;21(3):e0344480. doi: 10.1371/journal.pone.0344480 (PMC12974797; doi:10.1371/journal.pone.0344480)
Supplement: Attachment — Submitted filename: Comments.docx [file pone.0344480.s001.docx]

Overall Assessment

The topic is timely and relevant given the growing emphasis on multidisciplinary approaches in healthcare and the scarcity of data from Middle Eastern contexts. However, it would benefit from further refinement in the discussion, statistical interpretation, and contextualization of findings within broader global and regional literature.

Introduction

The introduction could better emphasize the research gap—why physiotherapists in medical wards (as opposed to ICUs or rehabilitation units) have been overlooked.

It could benefit from a clear statement of study objectives or hypotheses at the end.

“Therefore, this study aimed to assess physiotherapists’ knowledge, attitudes, and perceived barriers toward pressure injury prevention in Jordanian medical wards and identify associated demographic factors.”

Methods

Convenience sampling of 36 physiotherapists limits external validity; this limitation should be discussed earlier in the manuscript.

The statistical analysis section needs a brief mention of how missing data (if any) were handled.

Clarify whether the regression model controlled for confounders or if it was unadjusted.

Results

The results section sometimes repeats data already visible in tables. Summaries could be more interpretive rather than descriptive.

Ensure consistency between reported p-values (e.g., 0.00 should be written as p < 0.001).

Discussion

The discussion lacks a multidisciplinary systems perspective—for example, collaboration between physiotherapists, nurses, and wound care teams could be more deeply analyzed.

Broader international comparison (outside the Middle East) would strengthen the global relevance.

Recommend acknowledging limitations related to social desirability bias and self-reported measures.

The discussion section comprehensively interprets findings but lacks a broader multidisciplinary perspective linking physiotherapists’ preventive and rehabilitative roles to overall patient well-being. Incorporating literature highlighting the evolving preventive and psychosocial dimensions of physiotherapy would strengthen the scientific depth and contextual relevance of the discussion, emphasizing physiotherapists’ integral role in holistic pressure injury prevention. Add below phrase.

“The evolving role of physiotherapists extends beyond rehabilitation, encompassing preventive and psychosocial health dimensions [Nasif et al., 2025 <https://doi.org/10.31579/2578-8868/359> ]. Such multidisciplinary engagement supports patient-centered approaches essential in pressure injury prevention.”

Conclusion and Implications

The conclusion could benefit from more specific, evidence-backed recommendations (e.g., integrating PI prevention modules into physiotherapy curricula). Add a sentence emphasizing the study’s contribution to regional literature and how it can inform future interventional research.
