# Supplementary material for: “Healing hands: Understanding physiotherapists’ knowledge, attitudes and barriers on pressure injury prevention in the medical wards in Jordan”
Source: PLoS One. 2026 Mar 10;21(3):e0344480. doi: 10.1371/journal.pone.0344480 (PMC12974797; doi:10.1371/journal.pone.0344480)
Supplement: Attachment — Submitted filename: response file.docx [file pone.0344480.s003.docx]

**Response letter**

**PONE-D-25-37371**

**"Healing Hands: Understanding Physiotherapists' Knowledge, Attitudes and Barriers on Pressure Injury Prevention in the Medical Wards in Jordan"**

**PLOS ONE**

| comments | Response |
| --- | --- |
| Editor comments |  |
| . Please ensure that your manuscript meets PLOS ONE's style requirements, including those for file naming. The PLOS ONE style templates can be found at  https://journals.plos.org/plosone/s/file?id=wjVg/PLOSOne_formatting_sample_main_body.pdf and  [https://journals.plos.org/plosone/s/file?id=ba62/PLOSOne_formatting_sample_title_authors_affiliations.pdf](https://track.editorialmanager.com/CL0/https:%2F%2Fjournals.plos.org%2Fplosone%2Fs%2Ffile%3Fid=ba62%2FPLOSOne_formatting_sample_title_authors_affiliations.pdf/1/010f019a144b1ca3-3a5f1a51-021d-4d33-9325-9b9551895ba2-000000/zvf_jwXJROmwpQ5aEBQD0V3PmYyUNYoek4jxpB-7D9Y=234)    2. Thank you for stating the following financial disclosure: This research was funded by the Deanship of Scientific Research at King Khalid University; Grant number "RGP 2/263/46".  Please state what role the funders took in the study. If the funders had no role, please state: "The funders had no role in study design, data collection and analysis, decision to publish, or preparation of the manuscript." If this statement is not correct you must amend it as needed. Please include this amended Role of Funder statement in your cover letter; we will change the online submission form on your behalf.    3. Please amend the manuscript submission data (via Edit Submission) to include author Al-Ghabeesh S    4. Please amend your authorship list in your manuscript file to include author Suhair Al-Ghabeesh    5. Please include captions for your Supporting Information files at the end of your manuscript, and update any in-text citations to match accordingly. Please see our Supporting Information guidelines for more information: http://journals.plos.org/plosone/s/supporting-information.    6. If the reviewer comments include a recommendation to cite specific previously published works, please review and evaluate these publications to determine whether they are relevant and should be cited. There is no requirement to cite these works unless the editor has indicated otherwise.  7. Please review your reference list to ensure that it is complete and correct. If you have cited papers that have been retracted, please include the rationale for doing so in the manuscript text, or remove these references and replace them with relevant current references. Any changes to the reference list should be mentioned in the rebuttal letter that accompanies your revised manuscript. If you need to cite a retracted article, indicate the article’s retracted status in the References list and also include a citation and full reference for the retraction notice. | 1. Doe accordigly 2. "The funders had no role in study design, data collection and analysis, decision to publish, or preparation of the manuscript." included in the cover letter 3. Done and included 4. Done, missed by mistake (forgive) 5. Done , included in the data availability section, and it was highlighted as well 6. Yes , done 7. All references were checked one by one for retraction and no retraction were reported either on the journal page or google scholar, however I found one reference irrelevantly inserted and removed. Thank you for this important note. |
| Reviewer 1 | Response |
| Reviewer #1: Overall Assessment The topic is timely and relevant given the growing emphasis on multidisciplinary approaches in healthcare and the scarcity of data from Middle Eastern contexts. However, it would benefit from further refinement in the discussion, statistical interpretation, and contextualization of findings within broader global and regional literature. Introduction The introduction could better emphasize the research gap—why physiotherapists in medical wards (as opposed to ICUs or rehabilitation units) have been overlooked. It could benefit from a clear statement of study objectives or hypotheses at the end.  “Therefore, this study aimed to assess physiotherapists’ knowledge, attitudes, and perceived barriers toward pressure injury prevention in Jordanian medical wards and identify associated demographic factors.” | Thank you for your comments:  Added more global and international literature to both the introduction and the discussion  Introduction, the study aim was changed and we took your clearer statement. Thank you |
| Methods Convenience sampling of 36 physiotherapists limits external validity; this limitation should be discussed earlier in the manuscript. The statistical analysis section needs a brief mention of how missing data (if any) were handled. Clarify whether the regression model controlled for confounders or if it was unadjusted. | a. A convenient sample limitation had been added in the method section b. In the study, we collected data from all 36 physiotherapists targeted in the three hospitals. During data cleaning, it was confirmed that all 36 returned questionnaires were fully completed. We added a sentence to the "Statistical analysis" section to clarify this. c. The multivariate regression models were adjusted. As described in the "Statistical analysis" section and presented in Table 6, all independent variables (demographic factors: gender, age, marital status, monthly income, work experience, training, using guidelines, and unproportional providers) were entered simultaneously into the regression models for each of the three outcome variables. Statistical analysis section were modified accordingly. |
| Results The results section sometimes repeats data already visible in tables. Summaries could be more interpretive rather than descriptive. Ensure consistency between reported p-values (e.g., 0.00 should be written as p < 0.001). | Done. We have revised the results text for Table 5 to be more interpretive and less descriptive. We also corrected all 'P = 0.00' values to 'P < 0.001' in the text and tables for statistical consistency |
| Discussion The discussion lacks a multidisciplinary systems perspective—for example, collaboration between physiotherapists, nurses, and wound care teams could be more deeply analyzed.  Broader international comparison (outside the Middle East) would strengthen the global relevance. Recommend acknowledging limitations related to social desirability bias and self-reported measures.  The discussion section comprehensively interprets findings but lacks a broader multidisciplinary perspective linking physiotherapists’ preventive and rehabilitative roles to overall patient well-being. Incorporating literature highlighting the evolving preventive and psychosocial dimensions of physiotherapy would strengthen the scientific depth and contextual relevance of the discussion, emphasizing physiotherapists’ integral role in holistic pressure injury prevention. Add below phrase. “The evolving role of physiotherapists extends beyond rehabilitation, encompassing preventive and psychosocial health dimensions [Nasif et al., 2025 https://doi.org/10.31579/2578-8868/359 ]. Such multidisciplinary engagement supports patient-centered approaches essential in pressure injury prevention.” | Discussed further and highlighted the importance of the physiotherapist and the ref. were added or it's significant. |
| Conclusion and Implications The conclusion could benefit from more specific, evidence-backed recommendations (e.g., integrating PI prevention modules into physiotherapy curricula). Add a sentence emphasizing the study’s contribution to regional literature and how it can inform future interventional research. | added |
| Reviewer 2 | Response |
| Reviewer #2: Methods: Sample size: The rationale provided through GPower appears to be at odds with the final figure 36 participants. Determine if this represented the entire eligible population or if difficulties in recruitment restricted participation.  Instrument:  The process of adapting the questionnaire is clearly outlined, yet additional information is required about translation, cultural adjustments, and pretesting specific to the Jordanian setting. Data Analysis: While statistical tests like t-test, ANOVA, regression are appropriate, the assumptions normality, multicollinearity are not addressed. Think about adding confidence intervals and effect sizes for important findings. | Sample size: The sample size were the entire physiotherapist working in the three studied hospitals, as they were all interested in participating in the study; moreover, those physiotherapists were assigned to cover the entire hospital department as well. Therefore, During the study period, the number of physiotherapists meeting our eligibility criteria (working in medical wards at the three participating governmental hospitals) was finite. We therefore adopted a census approach and invited all eligible physiotherapists. The final sample of 36 represents the entire eligible population available at that time; consequently, the a-priori GPower calculation served only as initial planning guidance and was superseded by the census design. We have clarified this in the Methods.  Instruments  This was added as the questionnaire is written and administered in English. And a pilot study was conducted as well  We revised **Table 6** to report, for the **knowledge** and **attitude** outcomes, the **adjusted unstandardized coefficients (B)**, **95% confidence intervals**, and **standardized coefficients (β)**, alongside model fit statistics; p-values are consistently formatted. The **perceived barriers** model did not reach overall significance (F = 1.93, p = .09), so to avoid over interpretation we kept its presentation parsimonious in the main table; we are happy to add β and 95% CIs for barriers in a supplement if preferred. (See revised **Table 6**.) |
| Result: Explain the application of percentages compared to raw numbers to guarantee uniformity among tables. The regression analysis is clearly displayed but would improve by including standardized beta coefficients and confidence intervals. Certain p-values are presented as 0.00, they ought to be stated as < 0.001. | Across all descriptive tables, categorical variables are reported uniformly as count and percentage [n (%)]. Percentages are consistently calculated using the total sample size (N = 36) as the denominator because there were no missing data for the variables presented. |
| Discussion: Elaborate on the implications for policy and clinical training what targeted actions can organizations implement to enhance multidisciplinary pressure injury prevention? Add a brief section discussing how this research contributes to the current literature and suggest areas for future studies, such as intervention trials and larger national samples. Explicitly recognize the limitations of the small sample size, the singular setting, and the self-reporting bias. | Done, the recommendations were further elaborated focusing multidisciplinary approach in future studies for improving PI prevention  Sample size limitation was acknowledged and discussed in the limitations section |
| Result: Add a more emphatic statement linking enhanced knowledge and guideline application by physiotherapists to measurable patient-care results. Refrain from including new details or references to literature in this section | In the Results, we added a concluding sentence to the regression summary: “Overall, guideline use and prior PI training were associated with higher knowledge.” This emphasizes the observed relationship using only our data. |

Thank you
